# Smoking Abstinence Elicits Hyperalgesia: Neural Mechanism, Clinical Implications, and Pharmacological Managements

**DOI:** 10.1002/cns.71132

**Published:** 2026-09-10

**Authors:** Shan‐shan Hu, Chao‐feng Zhang, Battuvshin Lkhagvasuren, Di Wang

**Affiliations:** ^1^ Department of Laboratory Medicine, The First Affiliated Hospital of USTC, Division of Life Sciences and Medicine University of Science and Technology of China (USTC) Hefei China; ^2^ Department of Anesthesiology, The First Affiliated Hospital of USTC, Division of Life Sciences and Medicine University of Science and Technology of China (USTC) Hefei China; ^3^ Brain Science Institute Mongolian National University of Medical Sciences Ulaanbaatar Mongolia; ^4^ Department of Psychosomatic Medicine, Graduate School of Medical Sciences Kyushu University Fukuoka Japan; ^5^ Department of Forensic Medicine, School of Basic Medical Science Anhui Medical University Hefei China

**Keywords:** nicotine withdrawal, nociceptive sensitivity, pain/hyperalgesia, smoking abstinence

## Abstract

**Purpose:**

Nicotine exerts acute analgesic effects and serves as the major addictive component of tobacco. Nicotine withdrawal contributes to altered pain perception and hyperalgesia, further exacerbating postoperative pain and increasing opioid consumption in abstinent smokers during the perioperative period. This review aimed to systematically summarize the alterations in pain sensitivity induced by nicotine abstinence, elaborate the underlying neurochemical mechanisms, and clarify clinical intervention strategies as well as existing research limitations, so as to provide theoretical evidence for perioperative pain management in smoking cessation populations.

**Methods:**

This was a narrative review based on published animal and human studies. We comprehensively summarized changes in somatic sensitivity to cold, heat, and mechanical pain stimuli following nicotine abstinence. The clinical relevance of perioperative nicotine/tobacco abstinence for postoperative pain control was outlined. Moreover, we analyzed the regulatory roles of central neurotransmitters (serotonin, norepinephrine, endogenous opioids, and dopamine) and receptor activation remodeling in abstinence‐related pain sensitization. Additionally, the application value of nicotine replacement therapy in improving withdrawal‐induced hyperalgesia was summarized.

**Results:**

Nicotine abstinence significantly reduces pain threshold and enhances nociceptive sensitivity, leading to aggravated postoperative pain and elevated demand for opioid analgesics in perioperative smokers. Neurotransmitter dysfunction and abnormal receptor activation in the brain are core neural mechanisms mediating nicotine withdrawal‐induced hyperalgesia. Nicotine replacement therapy can effectively alleviate withdrawal‐evoked pain hypersensitivity and relieve its adverse impacts on postoperative pain outcomes. Current studies are limited by insufficient clinical data, especially studies focusing on chronic pain patients, while neuroimaging techniques exhibit great potential for exploring the neural mechanisms of abstinence‐related pain perception changes.

**Conclusions:**

Nicotine abstinence induces prominent pain sensitization via central neurochemical maladjustment. Nicotine replacement therapy is an effective pharmacological intervention for withdrawal‐induced hyperalgesia. This review deepens the systematic understanding of the mechanism underlying nicotine withdrawal‐altered pain sensitivity and provides feasible clinical strategies for perioperative pain management and smoking cessation intervention, supporting translational research and clinical practice in related fields.

## Graphical abstract

The cycle between nicotine use and pain modulation. Cigarette smoking leads to nicotine entering the body, which binds to nicotinic acetylcholine receptors (nAChRs) in the brain, triggering the release of different neurotransmitters, including dopamine (DA), norepinephrine (NE), serotonin (5‐HT), and GABA. This neurochemical cascade produces acute analgesic effects. However, repeated nicotine exposure causes nAChR desensitization as well as neurotransmitter alterations, resulting in nicotine dependence. Upon withdrawal, individuals experience hyperalgesia (increased pain sensitivity), which in turn intensifies cigarette craving and drives continued smoking behavior to alleviate discomfort, thereby perpetuating the cycle of addiction and pain dysregulation.

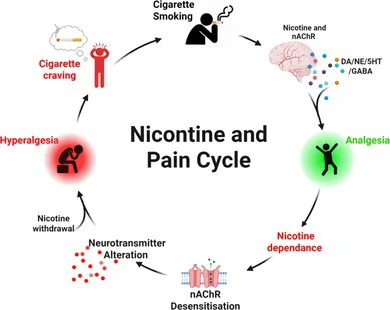

**Abbreviations**
CeA: central amygdala
CRF: corticotropin‐releasing factor
DRN: dorsal raphe nucleus
GABA: gamma‐aminobutyric acid
nAChRs: nicotinic acetylcholine receptors
VTA: ventral tegmental area

## 1 Introduction

Tobacco smoking, as a global health issue, has attracted considerable attention worldwide for its difficulty in quitting and high recurrence rate. In clinical practice, tobacco smoking is closely related to the increased risk of perioperative complications [1]. Therefore, tobacco smoking cessation for surgical patients is an important perioperative intervention to reduce anesthesia risks and improve postoperative recovery [2, 3]. Nicotine, the primary addictive substance found in cigarettes, has acute short‐term analgesic properties. However, the reduction in nicotine concentration due to tobacco deprivation has a considerable impact on nociceptive sensitivity in abstinent smokers. Compared to non‐smokers, regular smokers who temporarily abstain from tobacco often experience nociceptive hyperalgesia [4, 5, 6, 7]. These findings are relevant to perioperative pain management. The clinical studies have shown that alterations in pain tolerance in abstinent smokers result in worsened post‐surgical pain and increased demand for opioid analgesics [5, 8]. Therefore, understanding the mechanism of abstinence‐induced hyperalgesia is of great significance for developing scientific and reasonable management of post‐surgical pain in abstinent smokers. This narrative review summarizes the effects of nicotine withdrawal on pain perception, outlines the clinical significance of smoking abstinence‐induced hyperalgesia in perioperative care, and further examines the role of neurotransmitters in the supraspinal mechanisms underlying nicotine withdrawal‐induced hypersensitivity.

## 2 Smoking Abstinence Increases Pain Sensitivity due to Nicotine Withdrawal

The smoking prevalence in the general population has declined due to tobacco control worldwide. However, the cigarette smoking rates among individuals who suffer from persistent pain remain substantially higher (~24%–68%) [5, 9, 10, 11, 12]. In non‐smokers, nicotine delivered by cigarette smoke seems to produce transient analgesic properties [6, 13, 14]. By contrast, nicotine deprivation in abstinent smokers increases pain sensitivity, motivates the repeated use of nicotine/tobacco, and impedes smoking cessation [15, 16, 17]. These results seem complex but highlight the positive feedback mechanism of nicotine/tobacco, which reduces acute pain, further reinforcing smoking behavior.

Preclinical findings from rodent models demonstrate that nicotine withdrawal elicits transient and reversible hyperalgesia. For details, pain sensitivity tends to increase gradually over a period following withdrawal, then gradually, eventually returning to baseline [18, 19, 20, 21]. In a particular study [18], nicotine‐treated rats exhibited heightened pain sensitivity following withdrawal, with shorter latency for avoidance behaviors in response to electrical and thermal stimuli. Specifically, mechanical pain sensitivity rose significantly during the first 2 days before gradually decreasing, while thermal pain sensitivity increased over the first 4 days before returning to baseline at 7 days following nicotine withdrawal [18]. These findings suggest that rodents subjected to chronic nicotine also show decreased pain thresholds and increased hyperalgesia after withdrawal. Though tightly controlled animal experiments can unpack the underlying mechanisms in great detail, such work comes with clear drawbacks when translated to human clinical research. Since how the cerebral cortex processes pain differs widely between species, rodent models fail to mirror the full spectrum of emotional and cognitive pain responses seen in human smokers with persistent pain. This mismatch creates a major divide between lab findings and real‐world clinical use.

A variety of human experimental pain modalities have been adopted to assess pain reactivity in abstinent smokers and narrow the translational gap. Smoking abstinence increased pain sensitivity and decreased pain tolerance in smokers when subjected to various types of laboratory pain stimuli [7]. These stimuli include mechanical pain (such as von Frey filament tests and electrically induced mechanical pain), thermal pain (such as thermal radiation and contact thermistor tests), and cold pain (like immersion in cold water) [22, 23, 24, 25, 26, 27]. Specifically, young smokers experience heightened sensitivity to cold pain after quitting smoking compared to both non‐smokers and smokers who have not been deprived of nicotine [15, 25]. Interestingly, human studies also found that abstinent smokers exhibited higher sensitivity than non‐smokers after 48 h of withdrawal [26, 28]. Of note, this pain hypersensitivity has usually been reported in the acute/early phase of abstinence among regular smokers who temporarily abstain from tobacco cigarettes.

There are marked interspecies differences between rodents and humans in the central nervous system distribution of neurotransmitters and nicotinic acetylcholine receptors (nAChRs). While rodent models enable well‐controlled, high‐precision investigations into underlying mechanisms, their cortical circuits responsible for pain processing diverge substantially from human counterparts. Beyond this limitation, rodent studies fail to recapitulate the concurrent chronic pain, anxiety, and cognitive impairments commonly seen in human smokers. Taken together, these discrepancies mean preclinical observations cannot be directly extrapolated to clinical practice in humans.

## 3 Nicotine Deprivation Influences the Post‐Surgical Pain in Abstinent Smokers

Abstinence‐induced hyperalgesia has clinical implications for nicotine‐deprived smokers, such as post‐surgical pain management [7]. Post‐surgical pain is the most frequently examined condition of acute pain, with multiple studies reporting the impacts of smoking cessation on post‐surgical pain and opioid analgesics requirements (as shown in Table 1) [27, 29, 30, 31, 32, 33].

**TABLE 1:** Smoking cessation or nicotine replacement therapy on post‐surgical pain outcomes in abstinent smokers.

Study	PMID	Type and size	Country	Female (%)	Age (years)	Groups	Post‐surgical pain and opioids requirement
Woodside et al. 2000	11110060	Retrospective cohort study (*n* = 169)	China	100%	36.3 ± 5.3	(A) Nonsmokers	Group A had significantly lower opioids requirements compared to Groups B and C (*p* < 0.02)
						(B) Smoking abstinence (< 1 month)	
						(C) Current smokers	
Creekmore et al. 2004	15113988	Retrospective cohort study (*n* = 89)	United States	27.0%	65.0 ± 10.7	(A) Nonsmokers	Group A had significantly higher opioids requirements compared to Groups B (*p* = 0.023)
						(B) Current smoker (7 days abstinence)	
Shen et al. 2017	29121531	Retrospective cohort study (*n* = 148)	China	0%	49.9 ± 10.0	(A) Nonsmokers	Groups B had a higher opioids requirements after surgery than Group A (*p* < 0.05)
						(B) Smoking abstinence (< 1 month)	
Zhao et al. 2019	30653178	Prospective cohort study (*n* = 107)	China	0%	64.3 ± 2.3	(A) Nonsmokers	Group A had a higher opioids requirement compared with groups B and C (*p* < 0.05). Group B had a higher opioid requirements than group C
						(B) Smoking abstinence (< 3 weeks)	
						(C) (> 3 weeks)	
Kim et al. 2021	33574973	Retrospective cohort study (*n* = 144)	Korea	0%	57.9 ± 10.9	(A) Nonsmokers	Group B had significantly higher opioids requirements compared to Groups A and C (*p* < 0.001)
						(B) Nicotine abstinence (< 1 month)	
						(C) (> 1 month)	
Nazir. 2021	Not available	Prospective cohort study (*n* = 256)	Pakistan	Not mentioned	Not mentioned	Smoking cessation	Post‐surgical pain increased in patients with smoking cessation for 48 h, as compared with duration of cessation of more than 4 weeks
						(A) < 2 days	
						(B) 3–7 days	
						(C) 8 days to 4 weeks	
						(D) > 4 weeks	
Zhu et al. 2023	37132069	Prospective cohort study (*n* = 101)	China	0%	Not mentioned	(A) NRT (21or 42 mg/day)	Post‐surgical pain in the NRT group was significantly lower compared to placebo group (*p* < 0.001)
						(B) Placebo	
Maheshwari et al. 2024	39719676	Prospective cohort study (*n* = 100)	India	Not mentioned	Not mentioned	(A) NRT (21 mg/day)	NRT group had a lower post‐surgical pain compared to the placebo group (*p* < 0.05)
						(B) Placebo	
Olson et al. 2009	19923530	Prospective cohort study (*n* = 28)	United States	79%	45.2 ± 2.98	(A) Placebo	NRT groups had a higher post‐surgical pain than placebo group over the first hour after surgery (*p* < 0.01) and there was no difference between groups in the subsequent 5 days (*p* > 0.05)
						(B) NRT (5 mg/day)	
						(C) NRT (10 mg/day)	
						(D) NRT (15 mg/day)	

Abbreviation: NRT, nicotine replacement therapy.

Studies comparing abstinent smokers to non‐smokers consistently show that even short‐term abstinence worsens outcomes. Creekmore et al. [29] and Shen et al. [27] both found that abstinent smokers required significantly more opioids post‐surgery than non‐smokers. Notably, Woodside [32] reported that this effect was particularly pronounced in female smokers, who had significantly higher postoperative narcotic requirements than their non‐smoking counterparts, suggesting potential sex differences in withdrawal‐induced hyperalgesia. Estrogen and progesterone interact with the downregulation of nAChRs and dysfunction of the endogenous opioid system during nicotine withdrawal, which further impairs central pain modulation and makes female patients more vulnerable to severe postoperative hyperalgesia. Sex is an important biological variable in nicotine withdrawal‐related pain, which deserves more attention in clinical research. Critically, the duration of preoperative abstinence is a key modulator. A powerful, unified clinical argument emerges when synthesizing data from Nazir et al. [31], Kim et al. [30], and Zhao et al. [33]. These studies collectively define a distinct “vulnerability window” of acute nicotine withdrawal. Nazir directly demonstrated that patients who ceased smoking for only 48 h experienced significantly worse intraoperative hemodynamics and early postoperative pain compared to those abstinent for > 4 weeks. Zhao et al. found that patients abstaining for less than 3 weeks required more opioids than those abstaining for more than 3 weeks. Extending this, Kim et al. showed that patients abstinent for more than 1 month had significantly lower opioid requirements than those abstinent for less than 1 month. Taken together, these findings indicate that the peak of hyperalgesia occurs within the first few days to weeks of abstinence. Patients who quit smoking anywhere from 48 h up to 3 weeks prior to surgery fall right within this hyperalgesic peak window, resulting in the most severe postoperative pain. On the other hand, those who abstain from tobacco for 4 to 8 weeks or longer gain sufficient time for neural readjustment and recovery of endogenous pain regulatory pathways, which in turn brings postoperative pain outcomes back to normal levels.

Considering abstinence negatively impacts post‐surgical pain outcomes in smokers, nicotine replacement therapy (NRT) may serve as a primary recommendation for smoking cessation intervention [3]. Specifically, several clinical trials examined the effects of NRT, delivered as a nasal spray or skin patch, on post‐surgical pain management. For example, Zhu et al. [34] found that patients assigned to the NRT group that received 24‐h trans‐dermal nicotine patches (21, 42 mg) every day from the admission day until 48 h after surgery reported less postoperative pain among male smoking‐abstinent patients undergoing abdominal surgery. Similarly, a recent study conducted by Maheshwari et al. [35] corroborated the benefits of NRT on perioperative pain management under the condition of acute abstinence from smoking during hospitalization. One hundred abstinent tobacco smokers undergoing single‐level spinal fusion were randomized into placebo (*n* = 50) and NRT (*n* = 50) groups. Trans‐dermal nicotine patches (21 mg/24 h) were applied 24 h before surgery until 48 h after surgery and reduced postoperative pain and opioid requirements. Thus, the effects of NRT on post‐surgical pain in abstinent smokers may reflect the treatment of withdrawal rather than direct analgesic effects [6, 17].

However, it is contradictory that Luke and colleagues found a single administration of transdermal nicotine patch (5, 10, 15 mg) before anesthetic induction significantly increased postoperative pain scores and did not reduce the demand for morphine [36]. This apparent inconsistency, rather than indicating that NRT is ineffective, can be explained by critical differences in study design that inform clinical decision‐making.

First, the duration and continuity of NRT administration were significantly different. Positive tests for abdominal surgery and single‐segment spinal fusion nicotine patches were applied for at least 48 h (from 24 h before surgery to 48 h after surgery), maintaining stable plasma nicotine levels throughout the perioperative abstinence period. In contrast, Luke et al. used a pre‐induction patch, which was not continued after surgery, and may not be able to counteract the hyperalgesia caused by withdrawal, and may even produce a transient pro‐nociceptive effect through the acute activation of peripheral nicotinic acetylcholine receptors. Second, the timing of smoking cessation is crucial: positive results patients in the study actively abstained ≥ 24 h and experienced nicotine withdrawal, so NRT may exert its benefits by alleviating withdrawal symptoms. In Luke et al., the baseline withdrawal status was less clear; it may not be beneficial to give a single patch before withdrawal is fully developed. Third, surgical models and pain mechanisms are different: abdominal surgery and spinal fusion involve a large number of incisions, pain, and inflammation, in which hyperalgesia caused by withdrawal plays a major role. Luke et al.'s surgery (details not specified) may be less invasive, making any potential analgesic effect of NRT undetectable, while revealing its direct nociceptive properties. Fourth, gender differences may be important: Zhu et al. observed pain reduction only in male abstinent smokers, while Luke et al. did not report gender stratification results.

Taken together, these analyses showed that the different findings did not refute the utility of perioperative NRT, but emphasized that sufficient duration (≥ 48 h) and sufficient dose (≥ 21 mg/24 h), combined with confirmed preoperative abstinence, were important for achieving postoperative pain relief. A single low‐dose patch administered immediately before induction without continued delivery is unlikely to be effective and may even be harmful. Therefore, clinicians should adopt a continuous transdermal NRT protocol starting at least 24 h before surgery and lasting at least 48 h after surgery, especially for patients who have withdrawn or committed to perioperative withdrawal.

## 4 Neurotransmitter Dysfunction and Nicotine Abstinence‐Elicited Hyperalgesia

Nicotine exerts its pharmacological effects by interacting with nicotinic acetylcholine receptors (nAChRs), which are widely distributed across multiple brain regions, including the prefrontal cortex, primary sensorimotor cortex, thalamus, and hippocampus. These regions manage various brain functions but are closely involved in pain regulation in common. Consequently, nicotine may multidimensionally modulate nociceptive information. This complex processing likely engages multiple neurotransmitter systems, including serotonin, corticotropin‐releasing factor, endogenous opioid peptides, GABA, and dopamine (For details in Table 2) [37].

**TABLE 2:** Summaries of studies on central neurotransmitters underlie smoking cessation‐induced hyperalgesia.

Brain areas	Targets	Neurotransmitters	Species	PMID
Striatum; DRN; NRM	nAChRs↓	Serotonin↓	Rat	Nayak SV et al. 2000; Garduño J et al. 2012; Shen L et al. 2021
			Human	Slotkin TA et al. 2006
Unspecified	α4β2 nAChRs↓	Norepinephrine↓	Rat	Ramachandran Nair L et al. 2019; Li X et al. 2002
	α7 nAChRs↓		Human	Jorenby DE et al. 2006; J Gonzales D et al. 2006
CeA	CRF receptors	CRF	Rat	Lariviere WR et al. 2000; Cohen A et al. 2015
			Mice	Lariviere WR et al. 2000; Miguel TT et al. 2011
Striatum	Opioid receptors/G‐protein coupling↓	Met‐enkephalin	Mice	Isola R et al. 2000; Isola R et al. 2002
			Human	Nuechterlein EB et al. 2016
VTA; striatum	Dopamine receptors	Dopamine↓	Rat	Pistillo F et al. 2015; Ryu IS et al. 2017; Mao D et al. 2011; Zhang L et al. 2012; Mansvelder HD et al. 2002; Wood PB et al. 2008
			Mice	Pistillo F et al. 2015; Zhang L et al. 2012; Wood PB et al. 2008
			Human	Slotkin TA et al. 2006

Abbreviations: (↓), down‐regulation; CeA, central nucleus of amygdala; CRF, corticotropin‐releasing factor; DRN, dorsal rapheus nucleus; nAChRs, nicotinic acetylcholine receptors; NRM, nucleus raphe magnus; VTA, ventral tegmental area.

### 4.1 Serotonin

Serotonin can achieve analgesic effects by inhibiting the signal input of nociceptive stimuli. A decrease in serotonin levels in the brain can weaken the body's ability to suppress pain, increasing pain sensitivity. Both nAChR and serotonin receptors are distributed in the presynaptic nerve endings of DRN and striatum [38, 39]. Studies have shown that nicotine can enhance serotonin synaptic activity by activating nAChR on neurons, promoting serotonin release [38, 40]. During nicotine withdrawal, nicotine concentration in the brains of juvenile rats decreases, and serotonin synapses activity declines significantly, which may reduce serotonin release in the brain, weaken the body's ability to suppress pain, and increase pain sensitivity [41]. Therefore, nicotine withdrawal may lead to a diminished release of serotonin in the brain by decreasing the activation of nAChRs in the DRN and striatum, along with reducing serotonin synaptic activity, which ultimately results in heightened pain sensitivity in individuals experiencing nicotine withdrawal.

### 4.2 Norepinephrine and CRF

Like serotonin, a decrease in norepinephrine levels in the brain can increase the body's sensitivity to pain. Research has shown that nicotine can promote the release of norepinephrine by activating the α4β2 nAChR in the spinal cord and the α7 nAChR in the hippocampus. Studies indicate that during acute stress or anxiety, the body can produce analgesic effects by releasing CRF [42]. Microinjections of CRF have been shown to increase plasma norepinephrine levels. Further research by Miguel et al. demonstrated that activating the CRF system within the pain‐descending regulatory circuit may increase norepinephrine release, which can have a pain‐reducing effect [43]. Following nicotine withdrawal, rats display anxiety‐like behavior and heightened pain sensitivity. Injection of CRF antagonists into CeA can block both nicotine‐seeking behavior and increased pain sensitivity in these withdrawal‐induced rats [44]. In summary, nicotine withdrawal may impact the body's sensitivity to pain by reducing the activation of α4β2 nAChR and α7 nAChR, inhibiting the release of norepinephrine, or regulating the release of CRF and its receptor activation in the CeA.

### 4.3 Endogenous Opioids

The endogenous opioid system comprises opioid peptides, such as enkephalin and β‐endorphin, as well as opioid receptors. Enkephalin binds to μ‐opioid receptors and/or δ‐opioid receptors on interneurons [45]. Nicotine promotes the release of enkephalins in the striatum of mice by activating nAChR or facilitating the release of glutamate [46, 47]. Research indicates that after nicotine cessation, the density of μ‐opioid receptor binding sites in the basal ganglia and thalamus increases, along with a rise in enkephalin release in the nucleus accumbens [48, 49]. However, since opioid receptors function by binding to G proteins—which involves opioid receptor/G‐protein coupling—it may be misleading to assess functional changes in the endogenous opioid system solely by changes in enkephalin release or receptor binding site density [50, 51]. Some studies have shown that following nicotine cessation, there is impaired binding of the δ‐opioid receptor and κ‐opioid receptor, leading to reduced receptor activation [51]. These studies suggest that the degree of binding between opioid receptors and G proteins may be the leading indicator of whether the endogenous opioid system regulatory function is impaired. In summary, nicotine withdrawal may increase the body's pain sensitivity by regulating the release of enkephalins and activation of opioid receptors in the striatum and reducing the binding of opioid receptors to G proteins.

### 4.4 Dopamine

Research has demonstrated that nicotine withdrawal lowers dopamine levels in the nucleus accumbens and impairs the regulatory function of nAChR on dopamine release. This results in decreased sustained (tonic) and intermittent (phasic) dopamine release during withdrawal [52]. Dopamine is known to have analgesic effects through the activation of dopamine D2 receptors [53], and nicotine intake influences the midbrain VTA activity, leading to increased dopamine release in the striatum [54]. This may help explain why nicotine addicts exhibit significantly lower pain sensitivity compared to non‐addicts [24]. Additionally, dopamine released from the midbrain can bind to dopamine D1 receptors in the periaqueductal gray (PAG), which activates the pain descending inhibitory pathway and promotes the release of enkephalins, resulting in analgesic effects [55]. Studies utilizing animal models of nicotine withdrawal have indicated that nicotine withdrawal simultaneously diminishes the synaptic activity of both dopamine and serotonin in the striatum. Therefore, during nicotine withdrawal, the release of dopamine in the midbrain VTA and striatum of people with an addiction is reduced, and the activation of dopamine receptors is decreased, which may be one of the mechanisms underlying the increased pain sensitivity in nicotine withdrawal patients.

## 5 Peripheral Mechanisms of Abstinence‐Induced Hyperalgesia

Although supraspinal mechanisms are crucial for central pain modulation, nicotine withdrawal also exerts profound peripheral effects by directly sensitizing primary afferent nociceptors and lowering the pain threshold [15]. Under physiological conditions, peripheral C fibers—unmyelinated, slowly conducting primary afferent neurons responsible for transmitting noxious signals—are tonically inhibited by nicotinic acetylcholine receptors (nAChRs) located at their terminals. Nicotine maintains these neurons in a relatively quiescent state by modulating ion channel conductance and reducing excitability through activation of peripheral nAChRs [56], particularly the α7 and α4β2 subtypes [57].

Following abrupt cessation of smoking, the loss of this nAChR‐mediated inhibition leads to disinhibition of peripheral C fibers and pathological hyperactivity [58]. This manifests as spontaneous ectopic firing and exaggerated responses to noxious stimuli [56]. More importantly, this hyperactivity promotes anterograde (toward the spinal cord) and retrograde (toward peripheral tissues) propagation of action potentials along collateral branches of these C fibers. Retrograde impulse transmission [59], known as axonal reflexes, is a key driver of neurogenic inflammation.

Pathological retrograde discharge triggers rapid and robust release of preformed pro‐inflammatory neuropeptides stored in dense‐core vesicles within the terminal endings of C fibers. The two most abundant and characteristic of these are substance P (SP) and calcitonin gene‐related peptide (CGRP). SP released into peripheral tissues exerts multiple pro‐inflammatory effects: (1) it binds to neurokinin‐1 (NK‐1) receptors on postcapillary venules, inducing plasma extravasation and vasodilation, leading to local edema; (2) it directly stimulates mast cell degranulation, releasing histamine and other inflammatory mediators such as cytokines and proteases, further sensitizing nearby nociceptors—a process known as “peripheral sensitization;” (3) it promotes recruitment and activation of immune cells (e.g., neutrophils and macrophages), amplifying the local inflammatory cascade.

Meanwhile, CGRP—one of the most potent vasodilatory peptides—acts on vascular smooth muscle, causing sustained arteriolar dilation, increasing local blood flow, and resulting in the redness (erythema) and heat characteristic of inflammation. CGRP also enhances SP release and directly sensitizes TRPV1 (transient receptor potential vanilloid 1) and TRPA1 channels at nociceptor terminals [60], thereby lowering their thresholds for activation by heat, mechanical pressure, and chemical stimuli.

The cumulative consequence of this peripheral neurogenic inflammatory response is sensitization of the nociceptor terminals themselves‐an effect characterized by a leftward shift in the stimulus–response curve. This means that previously innocuous stimuli (such as light touch or mild warmth) now evoke pain, while noxious stimuli elicit exaggerated and prolonged pain responses. In surgical patients, the incision site provides an existing focus of tissue injury and inflammation; nicotine withdrawal‐induced neurogenic inflammation synergizes with surgical trauma to produce a “double‐hit” form of peripheral sensitization. This results in significantly lowered pain thresholds at the wound site, increased spontaneous pain, and heightened pain during movement, ultimately leading to higher postoperative pain scores and greater analgesic requirements among smokers who quit.

Thus, the increased pain sensitivity observed in individuals undergoing nicotine withdrawal is not merely a centrally driven perceptual change but is substantially amplified by a more inflamed, vasodilated, and sensitized peripheral wound environment. This peripheral sensitization offers additional opportunities for targeted peripheral interventions. Strategies such as local anesthetics, peripheral nerve blocks, or topical drugs that modulate neuropeptide release or receptor activity—such as NK‐1 receptor antagonists or CGRP receptor antagonists—could be explored as adjunctive approaches to mitigate the harmful consequences of acute nicotine withdrawal during the perioperative period.

## 6 Research Limitations and Future Directions

### 6.1 Study Subjects

Current evidence on the regulation of pain sensitivity by nicotine withdrawal is mainly conducted on rodents, and human studies on the mechanism underlying abstinence‐elicited hyperalgesia are relatively lacking. Due to the differences in the distribution of neurotransmitters and their receptors in the central nervous system of rodents, primates, and humans [61, 62], the direct application of findings from animal studies to humans is challenging. Recent advancements in multimodal brain imaging techniques have revealed that the psychological and physiological effects of nicotine withdrawal are associated with structural and functional abnormalities in specific brain regions [63, 64]. Combined with findings from animal studies, it's interesting to consider how to use neuroimaging in the future to explore the distribution of neurotransmitters, receptors, and brain regions that regulate pain sensitivity in abstinent smokers.

In addition, we also consider whether it is possible that more attention be paid to those regular smokers suffering from chronic pain in future human studies. Brain structure and function undergo adaptive changes in patients with chronic pain, and the neurons responsible for pain information processing gradually increase their responses to low‐intensity nociceptive stimuli. This adaptive process, termed “central sensitization,” can further disrupt the pain‐inhibiting pathways. In healthy individuals, activating these pain‐descending pathways typically results in analgesic effects [65]. However, these pathways can paradoxically lead to increased pain sensitivity in chronic pain. Therefore, future studies should examine how pain sensitivity changes in chronic pain patients after quitting nicotine, as this has significant scientific implications and practical value for improving clinical pain management strategies.

### 6.2 Smoking Cessation in Chronic Pain

Data suggest that motivation and intent to quit smoking are similar among smokers with and without chronic pain [66]. However, observational studies suggest that very few smokers entering chronic pain treatment successfully quit, even when offered efficacious tobacco intervention services [67, 68]. Thus, there is an urgent need for more research about how tobacco abstinence affects chronic pain and the development of effective methods to help smokers with chronic pain quit.

Consideration of the potential role of medical interventions to address smoking as a part of pain management raises several interesting issues [69]. Certainly, patients with chronic pain, like all other patients, would enjoy the dramatic benefits of smoking cessation on long‐term health. However, there are concerns to consider. The relationship between pain, smoking, and comorbid conditions such as depression and substance use disorders is complex, making it unclear how quitting tobacco may affect pain symptoms in both the short and long term. In the short term, since nicotine has acute analgesic effects, quitting smoking might initially worsen pain symptoms. Smokers often perceive nicotine as a means of managing stress and anxiety, and the withdrawal symptoms experienced during the process of quitting could complicate efforts to manage pain simultaneously. In the long term, it is possible that recovering from the effects of long‐term nicotine exposure may alleviate chronic pain, although this remains to be fully established. Using coping strategies other than smoking may enhance adaptive responses to chronic pain and improve overall functional status.

## 7 Conclusions

In summary, withdrawal alters pain sensitivity with a pattern of first increasing and then gradually returning to baseline levels, influenced by the type of pain stimulus experienced. This alteration in nociceptive perception has clinical relevance among patients who temporarily abstain from tobacco before surgery and leads to a post‐surgical phase of worse pain tolerance and more severe pain intensity. Research on substances use disorder indicates that many common addictive substance produce similar effects on pain sensitivity during withdrawal, such as opioid withdrawal [70], alcohol withdrawal [71], leading to hyperalgesia, and caffeine withdrawal leading to migraines [72] Therefore, nicotine withdrawal and other substance withdrawal may have partially similar neural mechanisms. While much of the focus in the mechanism studies is on the release of pain‐related neurotransmitters in the brain, it has also been proposed that nicotine deprivation could increase pain sensitivity at the peripheral level. This may occur due to vasodilation induced by the release of neuropeptides following activation of peripheral C‐fibers. Undoubtedly, future research exploring the underlying mechanisms will warrant increased attention to patients experiencing the acute phase of substance withdrawal, as well as the development of practical and effective interventions that can be easily integrated into clinical practice.

## Author Contributions

**Shan‐shan Hu** and **Chao‐feng Zhang:** compiled this manuscript; **Battuvshin Lkhagvasuren** and **Di Wang:** revised and reviewed this article.

## Funding

National Natural Science Foundation of China (No. 82471231) and Scientific Research Foundation of Anhui Province Education Department (Nos. 2023AH040395 and 2023AH053395).

## Ethics Statement

The authors have nothing to report.

## Consent

The authors have nothing to report.

## Conflicts of Interest

The authors declare no conflicts of interest.

## Data Availability

The data that support the findings of this study are available from the corresponding author upon reasonable request.
